# Protective and Detoxifying Effects of *Myrtus communis* Essential Oil Against Bisphenol A–Induced Metabolic Disturbances in Wistar Rats

**DOI:** 10.1155/bmri/1918173

**Published:** 2026-06-25

**Authors:** Mhimdi Mariem, Selmi Slimen, Sut Stefania, Dall′acqua Stefano, Sebai Hichem

**Affiliations:** ^1^ Laboratory of Functional Physiology and Valorization of Bio-Resources Higher Institute of Biotechnology of Beja, University of Jendouba, Jendouba, Beja, Tunisia, uj.rnu.tn; ^2^ Department of Pharmaceutical and Pharmacological Sciences, University of Padova, Padova, Italy, unipd.it

**Keywords:** *α*-pinene, bioavailability, BPA-metabolites, detoxification, *Myrtus communis*, SCFAs

## Abstract

Bisphenol A (BPA) is an endocrine disruptor widely used in industrial and consumer products. Its release into the environment raises major health concerns, particularly regarding metabolic disorders. After exposure, BPA leads to the accumulation of free BPA and its main metabolites, including bisphenol A‐glucuronide (BPA‐G), bisphenol A‐disulfate (BPA‐DS), and its chlorinated derivative, chlorinated bisphenol A‐diglucuronide (BPA‐DC). This study is aimed at evaluating the detoxifying effect of essential oil of *Myrtus communis* (EOMC) at 50, 100, and 200 mg/kg, and vitamin E (100 mg/kg), in male Wistar rats exposed to BPA (100 mg/kg). Results showed a significant decrease in serum levels of BPA and its metabolites, along with increased urinary excretion, indicating enhanced biotransformation and elimination. BPA exposure also elevated fecal short‐chain fatty acids (SCFAs) acetate, propionate, and butyrate, suggesting microbial dysbiosis and altered fermentation. EOMC and vitamin E treatments normalized SCFA profiles, demonstrating a modulatory effect on gut microbiota. The detection of *α*‐pinene and 1,8‐cineole in serum confirmed systemic bioavailability of EOMC and its role in detoxification. Overall, these findings highlight the protective effect of EOMC and vitamin E against BPA bioaccumulation and support their potential as natural detoxifying agents.

## 1. Introduction

Bisphenol A (BPA), scientifically referred to as 2,2‐bis(4‐hydroxyphenyl)propane, is a widely recognized endocrine‐disrupting compound found in the environment [1]. The chemical is copiously produced with a 5.3% annual growth rate in global demand and is widely applied in everyday products, such as packaging, electronics, medical equipment, and children′s toys [2, 3]. BPA is widely utilized in a variety of consumer products, including plastics, toys, cosmetics, and pharmaceuticals, leading to their ongoing release into the environment [4]. Consequently, these compounds have been identified across various environmental media such as wastewater, surface water, soil, sediments, and air [5]. BPA has been found not only in environmental samples, but also in specimens of human bodily fluids, such as urine, blood, and other fluids (amniotic fluid, follicle fluid, saliva, and breast milk) [6–8]. Even at low levels, prolonged exposure to bisphenols has been linked to several adverse health effects, including immunotoxicity, reproductive toxicity, metabolic disturbances, and the development of antimicrobial resistance [9, 10]. Following ingestion, BPA is mainly converted into bisphenol A‐glucuronide (BPA‐G) and bisphenol A‐disulfate (BPA‐DS) in humans. These metabolites are rapidly excreted via feces and urine, with a half‐life of less than 12 h. The majority of BPA absorbed by the intestine is likely subjected to glucuronidation primarily in the liver, with the resulting conjugate being predominantly excreted in bile [11, 12].

Liquid chromatography−tandem mass spectrometry (LC‐MS/MS) and gas chromatography−mass spectrometry (GC‐MS) have been used for the measurement of BPA and its metabolites (e.g., BPAG and hydroxylated‐BPA) in biological specimens [13–15]. For sample pretreatment, liquid–liquid extraction (LLE) have been used in the isolation and enrichment of BPA from solid or liquid matrices [16]. Because the analytical standards of BPAG, BPA mono‐(BPAS), and disulfate (BPA‐DS) are not commercially available, BPA analysis has, thus far, been focused on the measurement of “total BPA” [14, 17]. The conjugated forms of BPA (including BPA‐G and BPA‐DS) were estimated by subtraction of free BPA (analyzed without enzymatic deconjugation of samples) from total BPA (analyzed after deconjugation of samples by *β*‐glucuronidase) concentrations [14, 18].

Recent research has revealed that exposure to BPA not only disrupts endocrine functions but also profoundly alters the gut microbiota composition, leading to changes in the production of microbial metabolites such as short‐chain fatty acids (SCFAs), including acetate, propionate, and butyrate [19]. SCFAs are major fermentation products of dietary fibers by gut bacteria and play essential roles in maintaining intestinal homeostasis, energy metabolism, and immune balance [20]. Dysbiosis induced by BPA has been associated with excessive SCFA production, altered intestinal permeability, and metabolic disorders such as obesity and insulin resistance [21]. Therefore, modulation of gut microbial activity and SCFA levels represents an important mechanism by which natural antioxidants may counteract BPA‐induced metabolic toxicity [22].

Amid growing concerns about the toxicity of BPA, attention has shifted towards natural compounds that can mitigate its harmful effects. Among these, essential oils (EOs) are natural products extracted from plants and are well‐known for their diverse biological activities and therapeutic applications. Plant‐derived EOs are frequently used in pharmaceuticals for their antimicrobial, antioxidant [23, 24], antidiabetic, anticancer, and anti‐inflammatory properties [25, 26]. The EO of *Myrtus communis L.* has shown promising properties. This oil is particularly rich in terpenes, such as *α*‐pinene and limonene, as well as terpenoid oxides, with a notable concentration of 1,8‐cineole [27]. These bioactive components are known for their antioxidant [28, 29] and anti‐inflammatory effects [25, 30], making them potential candidates for reducing BPA‐associated toxicity.

In this study, we evaluated the protective effect of essential oil of *Myrtus communis* (EOMC), administered at different doses (50, 100, and 200 mg/kg BW), in *Wistar* rats exposed or not to BPA, including a cotreatment with vitamin E as an antioxidant reference. Indeed, free BPA and its metabolites were quantified in urine and serum by LC‐MS/MS. In parallel, oxidative stress parameters (malondialdehyde [MDA], superoxide dismutase [SOD], catalase [CAT], and glutathione peroxidase [GPx]) were assessed in serum. In addition, the bioavailability of the main compounds of the EO was analyzed in serum by GC‐MS, to better understand their systemic distribution and potential protective effect. Furthermore, SCFAs were determined in fecal samples by LC‐MS/MS, to explore the impact of BPA exposure and *M*. *communis* treatment on gut microbial metabolism and its potential link with systemic oxidative balance.

## 2. Material and Methods

### 2.1. Chemicals and Reagents

BPA (CAS 80‐05‐7 | Sigma‐Aldrich), benzanilide (CAS 93‐98‐1 | Sigma‐Aldrich), trichloroacetic acid (CAS 76‐03‐9 | Sigma‐Aldrich), 2‐thio‐barbituric acid (CAS 84030‐12‐6 | Sigma‐Aldrich), bovine catalase (PubChem CID: 135337101), epinephrine (PubChem CID: 5816), GSH (PubChem CID: 124886); methanol (PubChem CID: 18177619), ethyl acetate (CAS 141‐78‐6 | Sigma‐Aldrich), hexane (CAS 110‐54‐3 | Sigma‐Aldrich) were used in this experiment. Vitamin E and H_2_O_2_ were obtained in the form of commercially available from a pharmacy. All other chemicals used were of analytical grade.

### 2.2. Plant Collection

Myrtle leaves were harvested in March in the Hammam Bourguiba region (in the northwest of Tunisia) and identified by the botanist Chokri Hafsi.

### 2.3. EOs Preparation

The extraction method for the EO from *M*. *communis* leaves was hydrodistillation for 3 h using a Clevenger‐type apparatus. In summary, the plant material was submerged in water and brought to a boil, causing the EOs to evaporate along with the water vapor. The vapors were then condensed and collected. The distillate was subsequently isolated and dried over anhydrous sodium sulfate. The oil fractions were stored at 4°C until needed.

To ensure reproducibility, the extraction process was performed in triplicate independent preparations, and the lipid composition, including the relative abundance of major constituents, was analyzed for consistency across replicates.

### 2.4. GC‐MS Analysis

The extracted EOMC leaves was analyzed using GC‐MS using Trace GC ULTRA/Polaris Q (GC‐MS, Thermo Electron) by the method of Abidi et al., with slight modifications [31].

### 2.5. Acute Toxicity Study

The acute oral toxicity of EOMC was studied by WHO guidelines [32] for assessing the efficacy and safety of medicinal plants. EO of EOMC was orally administered to eight groups of mice (*n* = 10) at increasing doses (0, 50, 100, 200, 400, 600, 800, and 1000 mg/kg, *b*.*w*.). Animals were examined every 30 min for up to 4 h, then occasionally for a further 6, 8, and 12 h. After 24 h, mice were observed to record the appearance of mortality and signs of toxicity such as movement coordination disorders (ataxia), respiratory disorders (gasping), and cardiac problems (palpitations).

### 2.6. Male *Wistar* Rat and Treatment

#### 2.6.1. Animals and Experimental Design

In this experimental study, male Wistar rats were used, each weighing between 170 and 180 g and aged about 10 weeks. Animals were obtained from the Central Society of Pharmaceutical Industries of Tunisia (SIPHAT, Ben‐Arous, Tunisia) and maintained under standard conditions (22^°^C ± 0.5^°^C, 12/12 h light/dark cycle) with ad libitum access to food and water for a 2‐week acclimatization period.

Weight‐ and age‐matched animals were randomly assigned to eight groups, each consisting of six animals, and treated as follows [33]:

I. Corn oil, stripped of vitamin E, was administered as a vehicle to the control group at a dosage of 0.4 mL/kg orally for 30 consecutive days.
II. The BPA group received an oral 100 mg/kg of BPA for 30 consecutive days.
III. The EOMC group received an oral dose of 100 mg/kg of myrtle EO for 30 consecutive days.
IV. Myrtle 50 + BPA group received oral EOMC 50 mg/kg and BPA 100 mg/kg orally for 30 consecutive days.
V. Myrtle 100 + BPA group received oral EOMC 100 mg/kg and BPA 100 mg/kg orally for 30 consecutive days.
VI. Myrtle 200 + BPA group received oral EOMC 200 mg/kg and BPA 100 mg/kg orally for 30 consecutive days.
VII. Vitamin E group received oral 100 mg/kg of vitamin E (*α*‐*tocopherol)* for 30 consecutive days [34]
VIII. Vitamin E 100 + BPA group received oral vitamin E 100 mg/kg and BPA 100 mg/kg for 30 consecutive days.

To note, BPA was introduced into Groups IV, V, VI, and VII after the 7th day of treatment. In the cotreatment groups, EOMC was administered daily from Day 1. BPA was introduced from Day 7 so that rats received a 1‐week pretreatment with EOMC before BPA exposure. Both treatments were continued daily until the end of the 30‐day experimental period.

#### 2.6.2. Sample Collection

After 30 days of treatment, all rats were placed individually in metabolic cages for 24 h to collect their urine. Following this collection, the animals were fasted for 12 h and then sacrificed by decapitation. Blood was collected by intracardiac puncture, immediately centrifuged at 3000 rpm for 10 min at 4°C, and the separated serum was stored at −80°C until use.

### 2.7. Protective Effect of ***EOMC*** on BPA Detoxification: LC‐MS/MS Monitoring of Free BPA and Its Metabolites in Serum and Urine

#### 2.7.1. Extraction of BPA From Blood and Urine

Extraction of free BPA and its metabolites is performed by LLE, according to the method described by [35], with some modifications. Briefly, after thawing at room temperature, urine or serum (0.2 mL) was transferred into a 15‐mL polypropylene tube, and 2 ng/mL of benzanilide was added. The sample was extracted three times, each with 2 mL of ethyl acetate. The extracts were combined, washed with milli‐Q water, concentrated to near‐dryness under N2, and reconstituted with 0.2 mL of methanol before injection into LC‐MS/MS. The use of benzanilide as an internal standard ensured normalization of analyte responses, thereby minimizing matrix‐dependent variations between serum and urine and contributing to the consistent analytical performance observed across both biological matrices.

#### 2.7.2. LC‐MS/MS Analysis

The quantitative analysis of free BPA and its major metabolites (BPA‐G, BPA‐disulfate, and dichlorinated BPA) was carried out using a high‐performance liquid chromatography system coupled with a triple quadrupole tandem mass spectrometer (LC‐MS/MS), consisting of a Varian binary pump, autosampler, diode array detector, and a triple quadrupole MS320 (Varian/Agilent Technologies, Santa Clara, California, United States). Electrospray ionization (ESI) was performed in negative ion mode, using the multiple reaction monitoring (MRM) acquisition mode. Chromatographic separation was achieved using an Agilent XDB C18 column (3 × 150 mm, 3.5 *μ*m) maintained at 35°C. The mobile phase consisted of two solvents: eluent A (water with 0.1% formic acid) and eluent B (methanol with 0.1% formic acid). The elution gradient started with 90% A (10% B) held for 0.5 min, then shifted to 50% A/50% B, reaching 100% B at 10 min, and maintained isocratically until 20 min. The column was re‐equilibrated to 90% A from 20 to 25 min. The flow rate was set at 0.3 mL/min, and the injection volume was 10–20 *μ*L depending on the sample. Benzanilide was used as the internal standard to monitor extraction reproducibility, sample preparation, and instrumental variability throughout LC‐MS/MS analysis. Data acquisition was based on the following MRM transitions: BPA (227 > 212 and 227 > 133), BPA‐G (403 > 227), BPA‐sulfate (307 > 227), and dichlorinated BPA (295 > 201). The ESI source parameters were as follows: needle voltage 5000 V, drying gas temperature 300°C, drying gas pressure 22 psi, nebulizer pressure 55 psi, capillary voltage 40 V, and collision gas pressure (argon or nitrogen) at 1.5 mbar. The analytical method was validated according to commonly accepted bioanalytical validation guidelines. Calibration curves were established over the concentration range of 1–500 ng/mL and showed satisfactory linearity for all analytes (*R* ^2^ > 0.995). Limits of detection (LOD) and limits of quantification (LOQ) were determined using signal‐to‐noise ratios of 3 and 10, respectively. Extraction recovery was estimated by comparing analyte/internal standard peak area ratios obtained after extraction at different concentration levels. Matrix effects were evaluated using postextraction spiked samples compared with neat standard solutions. Intraday and interday precision were assessed at different concentration levels and expressed as relative standard deviation (%RSD). Detailed validation data for the LC‐MS/MS method are presented in Table 1.

**Table 1:** LC‐MS/MS method validation parameters for BPA and its metabolites in serum and urine.

Parameter	Matrix	BPA	BPA‐G	BPA‐DS	Cl‐BPA
**Linearity (** **R** ^2^ **)**	**Serum**	0.998	0.997	0.996	0.995
	**Urine**	0.997	0.996	0.995	0.994
**Calibration range (ng/mL)**	**Serum**	1–500	1–500	1–500	1–500
	**Urine**	1–500	1–500	1–500	1–500
**LOD (ng/mL)**	**Serum**	0.1	0.2	0.2	0.3
	**Urine**	0.12	0.20	0.22	0.35
**LOQ (ng/mL)**	**Serum**	0.3	0.5	0.5	1.0
	**Urine**	0.35	0.55	0.60	1.10
**Recovery (%)**	**Serum**	84.2 ± 5.8	88.5 ± 4.9	86.1 ± 6.2	81.7 ± 5.5
	**Urine**	86.5 ± 6.1	89.2 ± 5.3	87.0 ± 6.8	82.3 ± 5.9
**Matrix effect (%)**	**Serum**	92–107	90–105	88–110	91–108
	**Urine**	90–106	88–104	87–109	89–107
**Intraday precision (%RSD) (** **n** = 6**)**	**Serum**	4.5	5.1	5.8	5.3
	**Urine**	4.8	5.3	5.9	5.6
**Interday precision (%RSD) (** **n** = 6**)**	**Serum**	7.2	8.0	8.4	7.6
	**Urine**	7.5	8.3	8.6	8.0
**Accuracy (%)**	**Serum**	95–103	92–106	93–104	90–102
	**Urine**	94–102	93–105	92–104	91–101

### 2.8. Biochemical Evaluation of Oxidative Stress Markers in Serum

The serum was used to determine MDA and protein levels, as well as the activity of antioxidant enzymes, including the SOD, CAT, and GPx, as well as glutathione (in terms of total thiol groups) as a nonenzymatic antioxidant marker.

Protein concentrations were assessed using the Bradford method [36], with bovine serum albumin serving as a reference standard.

The lipid peroxidation levels in the testes and epididymis were assessed by measuring MDA concentration using the double heating method [37]. The molar extinction coefficient of the MDA‐TBA complex is *ε* = 1.592 × 105  M^−1^.cm^−1^. The results were expressed in nanomoles of malondialdehyde per milligram of protein.

The activity of SOD was determined using a modified epinephrine assay [38]. In an alkaline environment, the superoxide anion facilitates the auto‐oxidation of epinephrine to adrenochrome. Changes in absorbance were monitored at 480 nm. Enzyme activity is reported in units per minute per milligram of protein.

CAT activity was assessed by measuring the initial rate of hydrogen peroxide disappearance at 240 nm [39]. The reaction mixture consisted of 33 mM H_2_O_2_ in 50‐mM phosphate buffer at pH 7.0. The activity of CAT was determined using the extinction coefficient of 40 mM^−1^ cm^−1^ for H_2_O_2_.

The activity of GPx was determined using the Flohe and Gunzler method [40].

The total thiol group concentration was performed on testis and epididymis homogenates upon mixing with 0.25‐M Tris base and 20‐mM ethylenediaminetetraacetic acid (EDTA) at pH 8.2 [41]. The mixture was vortexed, and its absorbance was measured at 412 nm. The initial absorbance value was recorded as A1. Subsequently, 10 mM 5,5 ^′^‐dithiobis (2‐nitrobenzoic acid) (DTNB) was added. After an incubation time of 15 min, a new absorbance value, A2, was determined. The absorbance value of a white tube containing only DTNB and buffer was noted as B. The concentration of thiol groups (*μ*mol/mg of protein) per tube was calculated using the formula: (A2 − A1 − B) × 1.57 mM.

### 2.9. Serum Bioavailability Analysis of Key EOMC Compounds After 1 Month of Treatment

#### 2.9.1. Extraction of Compounds

The serum sample (100 mL) was put into a 1.5‐mL clean eppendorf tube; after that, 100 mL hexane and ethyl acetate (1:1) mixture was pipetted into the sample. The sample was shaken for 1 min to extract active compounds in a vortex mixer. Then, the sample was centrifuged in a high‐speed cryogenic centrifuge at 12000 rpm for 10 min, and the supernatant was transferred to a 1.5‐mL clean autosample tube. One microliter of the supernatant was injected into the GC‐MS system for analysis [42].

#### 2.9.2. GC‐MS Analysis

For the analysis, a Varian 3800 gas chromatograph was used, coupled with a Saturn 2000 T MS mass spectrometer using EI as the ionization source. For quantitative purposes, the mass spectrometer was set to work in MS/MS mode selecting m/z 91 as a precursor for monoterpenes and sesquiterpenoids; the selected ion was the m/z 231. The GC‐MS/MS method allowed the identification and quantification of the EOs′ main constituents, leading to measuring their serum bioavailability. The injector temperature was 225°C, the oven started at 55°C, stayed isothermal for 5.5 min, then increased to 250°C at 4°C/min and then to 280°C at 11°C/min.

### 2.10. Determination of SCFAs in Fecal Samples by LC‐MS

#### 2.10.1. Extraction of SCFAs

The extraction of SCFAs from fecal samples was carried out using a modified LLE protocol. Briefly, 30 mg of feces were accurately weighed and mixed with 300 *μ*L of OmniSolv ultrapure water. The mixture was homogenized for 20 s at 6500 rpm using a Precellys 24 tissue homogenizer, and this step was repeated three times to ensure complete dispersion. The suspension was then incubated at 4°C for 30 min under gentle agitation, followed by centrifugation at 13,000 g for 30 min to obtain the supernatant. An aliquot of 100 *μ*L of the fecal supernatant was transferred into a 0.6‐mL microtube containing 10 *μ*L of 5 M HCl to adjust the pH to approximately 2, ensuring SCFA stabilization. The acidified sample was then extracted with 100 *μ*L of anhydrous diethyl ether by vertexing and incubating on ice for 5 min. After centrifugation at 10,000 g for 5 min, the upper organic phase (diethyl ether layer) containing SCFAs was carefully collected and transferred into a clean microtube containing anhydrous sodium sulfate to remove residual water. The extraction was repeated twice under identical conditions to maximize SCFA recovery, and all diethyl ether phases were combined for subsequent LC‐MS/MS analysis.

#### 2.10.2. LC‐MS Analysis

An LC‐MS/MS system consisting of an Agilent 1290 HPLC (Agilent Technologies, Glostrup, Denmark) coupled to a QTRAP 5500 triple quadrupole mass spectrometer (AB Sciex, Framingham, Massachusetts, United States) was used for SCFA analysis, as previously described by [43]. The method validation parameters are summarized in Table 2.

**Table 2:** LC‐MS/MS method validation parameters for short‐chain fatty acids (SCFAs) in fecal samples.

Analyte	Linearity *R* ^2^	Intraday RSD % (L/M/H)	Interday RSD % (L/M/H)	Accuracy % (L/M/H)	LOD (*μ*g/mg)	LOQ (*μ*g/mg)
Acetate acid	0.972	10.5/6.2/3.5	14.2/8.1/5.6	95.2/98.6/101.3	0.188	0.57
Propionate	0.983	8.1/5.4/3.2	11.0/7.9/6.1	96.5/100.2/103.1	0.098	0.30
Butyrate	0.985	6.5/4.3/2.8	9.2/6.7/5.4	97.8/101.5/104.2	0.030	0.092

### 2.11. Statistical Analysis

All results were presented as mean ± SD. Statistical analysis was performed using one‐way analysis of variance (ANOVA) with GraphPad Prism statistical software, Version 9.0.2 (GraphPad Software Inc., La Jolla, California, United States), to compare between groups. When significant differences were found, post hoc comparisons were performed using Tukey′s HSD to correct for multiple testing. *p* values less than 0.05 were considered statistically significant.

## 3. Results

### 3.1. Chemical Composition of EOMC

The composition of EOMC with the retention times of the compounds are presented in Table 3. The most abundant constituents present in this oil were alpha‐pinene (59.749%), 1.8‐cineole (18.651%), and dI‐limonene (7.020%) [44].

**Table 3:** Composition of essential oil of *Myrtus communis* L.

Peak	Compounds	% of total	RT (min)
1	Alpha.‐thujene	0.618%	6.009
2	Alpha.‐pinene	59.749%	6.197
3	Beta.‐pinene	0.410%	7.111
4	Butanoic acid, 2‐methyl‐, 2‐methylpropyl ester	0.260%	7.674
5	l‐phellandrene	0.450%	7.739
6	Delta.3‐carene	0.768%	7.879
7	p‐cymene	2.297%	8.214
8	dl‐limonene	7.020%	8.337
9	1,8‐cineole	18.651%	8.393

### 3.2. Toxicity Test

No visible signs of toxicity or deaths were observed in animals treated with EOMC at increasing doses (50–1000 mg/kg). The effects of acute oral EOMC treatment are summarized in Table 4. In acute toxicity tests, oral administration of EOMC to male rats did not cause any significant alterations in behavior, respiration, sensory responses of the nervous system, or gastrointestinal effects during handling. In addition, no mortality or toxic reactions were observed in any animal in any group after 72 h of administration. The LD_50_ is therefore greater than 1000 mg/kg body weight for EOMC.

**Table 4:** Acute oral toxicity assessment of essential oil of *Myrtus communis* (EOMC) in mice at different doses.

Treatments (mg/kg b.w.)	Number of animals	Visible signs of toxicity
0	6	None
50	6	None
100	6	None
200	6	None
400	6	None
600	6	None
800	6	None
1000	6	None

*Note:* Clinical signs of toxicity were assessed based on standard observational criteria, including tremors, piloerection, salivation, lethargy, convulsions, abnormal posture, and altered locomotor activity.

### 3.3. Evaluation of the Role of EOMC and Vit E in the Detoxification and Excretion of BPA and Its Metabolites via Serum and Urinary Matrices

**Figure 1:**
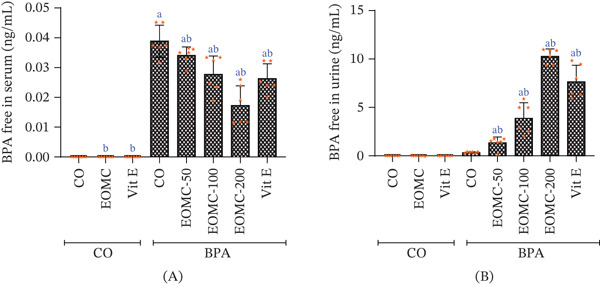
Effect of EOMC and vitamin E on free bisphenol A levels in (A) serum and (B) urine in rats exposed to BPA. BPA, bisphenol A; CO, corn oil (control group); EOMC, essential oil of *Myrtus communis*; Vit E, vitamin E. EOMC‐BPA groups received EOMC at 50, 100, and 200 mg/kg in combination with BPA; Vit E‐BPA group received vitamin E (100 mg/kg) with BPA. Statistical analysis was performed using one‐way ANOVA followed by Tukey′s post hoc test. Data are expressed as mean ± SD (*n* = 6 rats/group).^a^ *p* < 0.05 versus control group; ^b^ *p* < 0.05 versus BPA group.

**Figure 2:**
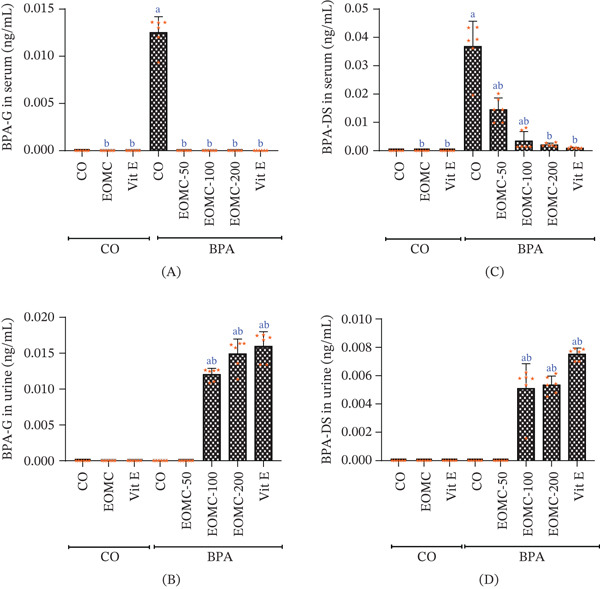
Effect of EOMC and Vit E on BPA‐glucuronide (BPA‐G) and BPA‐disulfate (BPA‐DS) levels in (A, C) serum and (B, D) urine of Wistar rats exposed to BPA. BPA, bisphenol A; CO, corn oil (control group); EOMC, essential oil of *Myrtus communis*; Vit E, vitamin E. EOMC‐BPA groups received EOMC at 50, 100, and 200 mg/kg in combination with BPA; Vit E‐BPA group received vitamin E (100 mg/kg) with BPA. Statistical analysis was performed using one‐way ANOVA followed by Tukey′s post hoc test. Data are expressed as mean ± SD (*n* = 6 rats/group). ^a^ *p* < 0.05 versus the control group; ^b^ *p* < 0.05 versus BPA group.

**Figure 3:**
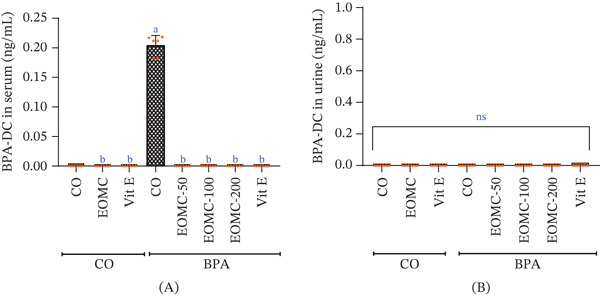
Effect of EOMC and Vit E on chlorinated BPA‐diglucuronide (BPA‐DC) levels in (A) serum and (B) urine in rat exposed to BPA. Number of rats: *n* = 6/group. BPA, bisphenol A; CO, corn oil (control group); EOMC, essential oil of *Myrtus communis*; Vit E, vitamin E. EOMC‐BPA groups received EOMC at 50, 100, and 200 mg/kg in combination with BPA; Vit E‐BPA group received vitamin E (100 mg/kg) with BPA. Statistical analysis was performed using one‐way ANOVA followed by Tukey′s post hoc test. Data are expressed as mean ± SD (*n* = 6 rats/group). ^a^ *p* < 0.05 versus control group; ^b^ *p* < 0.05 versus BPA group.

LC‐MS analysis of free BPA (Figure 1), its metabolites (BPA‐G and BPA‐disulfate) (Figure 2), and its chlorinated derivative (dichlorobisphenol) (Figure 3) in serum and urine samples collected at the end of the experiment. In rats in the BPA‐only group, a significant accumulation of free BPA and its metabolites was observed in the serum, whereas these compounds were absent or below the detection limit in urine. In contrast, rats pretreated with EOMC, regardless of the dose administered (50, 100, or 200 mg/kg), as well as those treated with vitamin E, showed an inverse profile: Concentrations of free BPA and its metabolites were significantly reduced in serum but present in urine, indicating improved renal elimination of BPA and its derivatives.

### 3.4. Effect of EOMC and Vit E on Serum Redox State

The impact of different exposure conditions on serum lipid peroxidation, assessed by MDA assay, is presented in Table 5. Vitamin E treatment resulted in decreased serum lipid peroxidation levels compared with the BPA‐exposed group. As expected, BPA administration induced a significant increase in serum MDA concentrations compared with the control group. Coadministration of EOMC significantly reduced MDA levels, even at the lowest dose administered (50 mg/kg).

**Table 5:** Effect of EOMC and Vit E on oxidative stress markers in serum in rats exposed to BPA.

	MDA (nmol/mg protein)	SOD (U/mg protein)	CAT (nmol H_2_O_2_/min/mg protein)	GPx (nmol GSH/min/mg protein)	Thiols groups (*μ*mol/mg protein)
Control	151.72 ± 1.99	12.07 ± 1.56	34.66 ± 2.45	28.55 ± 0.78	44.21 ± 8.47
EOMC	149.454 ± 2.21^b^	23.38 ± 0.89^ab^	20.54 ± 1.74^ab^	26.72 ± 0.66^ab^	30.17 ± 7.83^a^
Vit E	123.87 ± 4.15^ab^	28.54 ± 3.08^ab^	33.98 ± 1.55^b^	23.17 ± 1.11^ab^	26.55 ± 2.34^a^
BA	434.74 ± 2.41^a^	7.57 ± 1.89^a^	11.62 ± 1.43^a^	5.74 ± 0.23^a^	22.56 ± 4.06^a^
EOMC − 50 + BPA	291.44 ± 13.02^ab^	8.46 ± 1.21	12.95 ± 1.12^a^	9.28 ± 0.15^ab^	22.62 ± 2.82^a^
EOMC − 100 + BPA	255.73 ± 2.06^ab^	14.30 ± 1.99^b^	16.67 ± 1.33^ab^	16.22 ± 0.18^ab^	23.34 ± 2.85^a^
EOMC − 200 + BPA	152.22 ± 8.74^b^	17.93 ± 1.68*a* ^b^	19.34 ± 2.23^ab^	23.94 ± 0.73^ab^	25.13 ± 3.75^a^
Vit E + BPA	269.71 ± 4.78^ab^	20.56 ± 3.20^ab^	23.54 ± 2.16^ab^	16.96 ± 1.40^ab^	24.21 ± 3.17^a^

*Note:* Number of rats: *n* = 6/group. Control: negative control group, BPA: bisphenol A, EOMC‐100: essential oil of *Myrtus communis*, EOMC‐BPA: essential oil of *Myrtus communis* at (50,100, and 200 mg/kg) with bisphenol A, Vit E‐BPA: vitamin E with bisphenol A, Vit E: vitamin E. Values are the mean ± SD.

^a^ *p* < 0.05 compared with the control group.

^b^ *p* < 0.05 compared with BPA group (ANOVA test).

The effect on SOD, CAT, GPx activities, and total thiol content was also investigated (Table 5). Compared with sham controls, SOD activity was strongly and significantly attenuated by BPA exposure, and a dose‐dependent recovery was observed when EOMC was coadministered. Interestingly, the enzyme activities were almost restored to control levels. Similar results were observed for CAT and GPx activities. On the other hand, for total thiol content in serum matrices, a significant reduction was observed upon BPA exposure compared with sham controls, but it was significantly restored by EOMC at 200 mg/kg.

### 3.5. Serum Distribution of the Major Bioactive Constituents of EOMC

**Figure 4:**
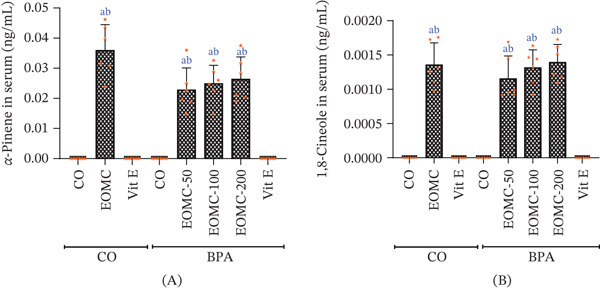
Serum concentrations of two major compounds of *Myrtus communis* essential oil in Wistar rats: (A) *α*‐pinene and (B) 1,8‐cineole, after oral administration of EOMC alone or in coexposure with bisphenol A (BPA). BPA, bisphenol A; CO, corn oil (control group); EOMC, essential oil of *Myrtus communis*; Vit E, vitamin E. EOMC‐BPA groups received EOMC at 50, 100, and 200 mg/kg in combination with BPA; Vit E‐BPA group received vitamin E (100 mg/kg) with BPA. Statistical analysis was performed using one‐way ANOVA followed by Tukey′s post hoc test. Data are expressed as mean ± SD (*n* = 6 rats/group). ^a^ *p* < 0.05 versus control group; ^b^ *p* < 0.05 versus BPA group.

The bioavailability of the main bioactive molecules of EOMC was analyzed in rat serum using gas chromatography. The results show that *α*‐pinene (Figure 4A) and 1,8‐cineole (Figure 4B) are absent in the control (CO), BPA alone, and vitamin E groups, but are detected at significant concentrations in the serum of rats treated with the EO, alone, or in association with BPA. Indeed, in all EO‐treated groups, *α*‐pinene showed a serum concentration approximately three times higher than that of 1,8‐cineole, thus highlighting its superior bioavailability.

### 3.6. Effect of EOMC and Vit E in Restoring SCFA Balance Altered by BPA Intoxication

**Figure 5:**
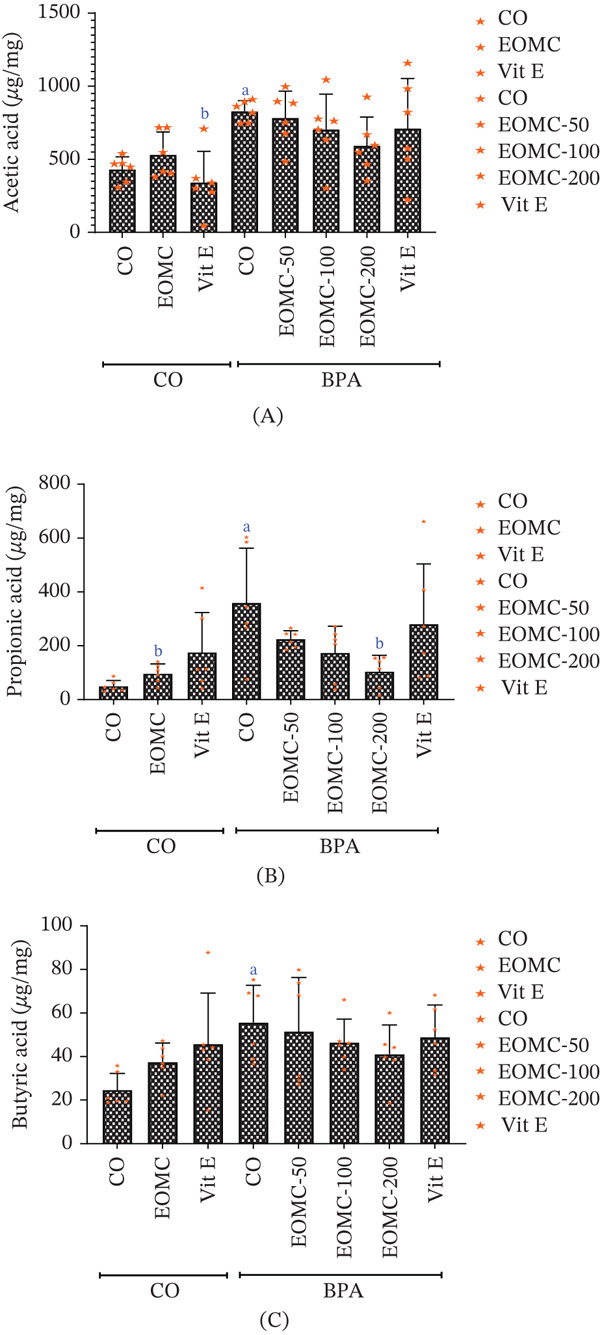
Effect of EOMC and Vit E in restoring SCFA balance altered by BPA intoxication: (A) acetic acid, (B) propionic acid, and (C) butyric acid. BPA, bisphenol A; CO, corn oil (control group); EOMC, essential oil of *Myrtus communis*; Vit E, vitamin E. EOMC‐BPA groups received EOMC at 50, 100, and 200 mg/kg in combination with BPA; Vit E‐BPA group received vitamin E (100 mg/kg) with BPA. Statistical analysis was performed using one‐way ANOVA followed by Tukey′s post hoc test. Data are expressed as mean ± SD (*n* = 6 rats/group). ^a^ *p* < 0.05 versus control group; ^b^ *p* < 0.05 versus BPA group.

The concentrations of the main SCFAs are shown in figure such as acetic (Figure 5A), propionic (Figure 5B), and butyric (Figure 5C) were quantified in the feces of rats from different experimental groups. Indeed, BPA exposure resulted in a significant increase in fecal concentrations of acetic, propionic, and butyric acid compared with the control group (*p* < 0.05), reflecting an alteration of intestinal microbial metabolism induced by BPA. However, preventive treatment with EOMC (50, 100, and 200 mg/kg) and Vit E showed a decrease in the concentration of acetic and butyric acid without statistical difference. Interestingly, the concentration of acetic acid in the vitamin E alone group showed a significant decrease compared with the BPA group. Whereas for the concentration of propionic acid, we notice a significant prevention with the 200 mg/kg dose of EOMC.

## 4. Discussion

Our study highlights the potential preventive and detoxifying role of EOMC against BPA‐induced toxicity and bioaccumulation in male *Wistar* rats. It is important to note that the BPA dose used in the present study (100 mg/kg/day) is considerably higher than typical human environmental exposure levels. The current tolerable daily intake (TDI) established by the European Food Safety Authority is 0.2 ng/kg/day, based on immune‐related effects (updated value) [45], whereas earlier NOAEL values were reported at 5 mg/kg/day [46]. Therefore, the administered dose largely exceeds these thresholds, including both NOAEL and reported LOAEL values, and should be considered as a high‐dose toxicological model. This experimental design was intentionally adopted to induce clear and reproducible metabolic and oxidative disturbances within a short exposure period [44, 47]. This high‐dose approach is commonly used in experimental toxicology to reliably induce measurable biological effects within a limited time frame. From a toxicokinetic perspective, BPA is rapidly metabolized and eliminated through glucuronidation and sulfation pathways, resulting in transient systemic levels at low environmental doses [13, 48]. Therefore, administering 100 mg/kg/day allows for detectable concentrations of BPA and its metabolites in serum and urine, which is essential for investigating metabolic disturbances, oxidative stress, and the protective effects of EOMC. Nevertheless, caution should be exercised when extrapolating these findings to human exposure scenarios, as dose–response relationships at environmentally relevant low doses may differ substantially. Future studies using lower, environmentally relevant BPA doses are warranted to confirm the translational relevance of these results.

To achieve this, we initially utilized GC‐MS to highlight the chemical composition of the EO, revealing two primary bioactive molecules: *α*‐pinene and 1,8‐cineole. The results provide strong evidence that coadministration of EOMC with BPA reduces oxidative stress, suggesting increased metabolic transformation and elimination. This protective effect is further supported by our biochemical and metabolic findings. In fact, rats exposed only to BPA, free BPA, BPA‐G, BPA‐DS, and dichlorobisphenol A (Cl₂‐BPA) accumulate in serum and are almost absent in urine. In contrast, in rats treated with EOMC, these compounds decrease in blood and reappear in urine, suggesting activation of conjugation pathways (Phase II) and enhanced renal elimination. Indeed, recent studies have shown that BPA‐G is biologically active, capable of promoting adipogenesis and lipid accumulation in human and murine preadipocytes [49]. Moreover, BPA‐G and BPA‐DS have also been described as proadipogenic, inducing the differentiation of white adipocytes while inhibiting that of brown ones [50]. These chlorinated derivatives exhibit higher estrogenic activity than nonchlorinated BPA. Indeed, 3,3 ^′^‐dichlorobisphenol A (3,3 ^′^‐diClBPA) has been shown to have a higher affinity for the estrogen receptor alpha (ER*α*) than BPA, and to induce an increase in uterine weight in ovariectomized rats, indicating enhanced estrogenic activity in vivo [51].

Furthermore, Mutou et al. reported that 3,3 ^′^‐diClBPA more intensely stimulates estradiol response systems in vitro, through a GFP/ERE expression assay, thus confirming its strong endocrine disrupting potential [52]. Furthermore, the detection of these compounds in humans by Andra et al. demonstrates real exposure and potential risks on the energy and metabolic balance [53]. Our results reveal that EOMC promotes the elimination of BPA and its conjugates via the activation of biotransformation pathways (Phase II) and excretion transporters. Several studies confirm that monoterpenes present in EOs can stimulate these mechanisms. Previous studies have suggested that *α*‐pinene and 1,8‐cineole can activate Nrf2 signaling and induce Phase II enzymes such as UGT and GST [54, 55]. Although these mechanisms may help explain the reduction of BPA metabolites and oxidative stress observed in our study, we did not directly measure Nrf2 or enzyme activity. Therefore, these interpretations remain hypothetical. In fact, in vivo study revealed that *Citrus aurantium* extracts (rich in limonoids) activate GST and quinone reductase (QR) in the liver and intestine, showing the strengthening of detoxification pathways [56]. This power to modulate the metabolic profile of BPA could also clarify the reduction in oxidative stress observed in cotreated rats, reflecting a synergy between the mechanisms of detoxification and enzymatic defense. Indeed, BPA results in severe systemic oxidative stress, as indicated by a marked increase in serum MDA, as well as a significant decrease in the levels of antioxidant enzymes (SOD, CAT, and GPx) and thiol groups. This disruption of the oxidative profile is consistent with the literature showing that BPA disrupts the redox balance by increasing lipid peroxidation and inhibiting endogenous antioxidant enzymes [57, 58]. In contrast, groups treated with EOMC or vitamin E alone showed an overall improved antioxidant status. EOMC alone increased the activity of antioxidant enzymes while maintaining MDA levels comparable with the CO, demonstrating its ability to strengthen antioxidant defenses without inducing oxidative stress. Interestingly, EOMC administered with BPA dose‐dependently attenuates the toxic effects of the latter. From 50 mg/kg, a significant decrease in MDA is observed, with a progressive restoration of SOD, CAT, and GPx. At a dose of 200 mg/kg, these parameters are almost equal to those observed in the control group, which proves a certain systemic protective effect. This antioxidant potential of EOMC is comparable, or even superior, to that of vitamin E in the model studied. These results confirm previous data according to which the major monoterpenes of EOMC, such as *α*‐pinene and 1,8‐cineole, possess potent antioxidant properties and can intervene effectively in contexts of induced oxidative stress [55, 59]. All these observations position EOMC as a promising natural agent to mitigate chronic oxidative stress caused by environmental pollutants such as BPA.

Moreover, the detection of certain bioactive compounds of EOMC in serum is of particular interest, as it provides evidence of their in vivo absorption following oral administration, as assessed by GC‐MS. In the present study, *α*‐pinene and 1,8‐cineole were detected in rat serum at the end of the experimental period, indicating that these compounds or their residual levels persist in the systemic circulation after repeated administration of EOMC. These findings are consistent with previous studies reporting that *α*‐pinene and 1,8‐cineole can be absorbed and detected in biological fluids following oral administration [60, 61].

However, it is important to note that the detection of these compounds reflects systemic exposure rather than their pharmacodynamic relevance. Therefore, their direct contribution to the observed protective effects of EOMC cannot be established and remains hypothetical. Although previous studies have reported various biological activities of these monoterpenes, such effects were not directly assessed in the present study.

In addition, the persistence of these compounds in serum despite BPA exposure suggests that their systemic presence is maintained under the experimental conditions; however, no definitive conclusion can be drawn regarding potential interactions between BPA exposure and their pharmacokinetic behavior.

SCFAs are the end products of intestinal microbiota metabolism. They contribute to maintaining the integrity of the intestinal mucosa, improve glucose and lipid metabolism, control energy expenditure, and regulate the immune system and inflammatory responses [62]. Regarding the effect of BPA on metabolism, our results demonstrate that BPA exposure significantly increased fecal levels of acetate, propionate, and butyrate. These changes may reflect an alteration in intestinal microbial fermentation activity. A similar increase in these SCFAs has been reported by [63] following BPA exposure. However, it is important to note that elevated SCFA levels are not necessarily indicative of a pathological condition and may depend on the physiological and microbial context. The preventive administration of EOMC, particularly at a dose of 200 mg/kg, was associated with SCFA levels closer to those of the control group, suggesting a potential modulatory trend on intestinal metabolic profiles rather than a direct normalization effect. This effect could potentially be related to the antimicrobial and prebiotic properties of monoterpenes such as *α*‐pinene and 1,8‐cineole, which have been reported to influence gut microbial activity and intestinal redox balance [64, 65]. However, since no direct analysis of gut microbiota composition (e.g., 16S rRNA sequencing) was performed in the present study, these interpretations remain indirect and should be considered as hypothetical. The reduction in excessive levels of SCFAs, associated with the decrease in BPA and its metabolites in the serum, indicates that EOMC could act both by restoring the integrity of the intestinal barrier and by promoting the detoxification of BPA through the enterohepatic and renal pathways.

This study has some limitations that should be acknowledged. The BPA dose used (100 mg/kg/day) is much higher than environmentally relevant human exposure, which limits direct extrapolation to real‐life conditions despite its usefulness inducing clear toxic effects. In addition, no pharmacokinetic analysis was performed for *α*‐pinene and 1,8‐cineole, and single‐time‐point sampling did not allow determination of Cmax, Tmax, or AUC, limiting interpretation of their kinetic behavior. The proposed molecular mechanisms of EOMC were not directly investigated, as no Nrf2 or Phase II enzyme measurements were performed, and therefore remain hypothetical. Moreover, gut microbiota composition was not analyzed, so changes in SCFAs cannot be directly linked to microbial alterations. Finally, normality testing was not performed prior to statistical analysis, and minor matrix effects may have influenced some LC‐MS/MS parameters.

In summary, these results highlight the importance of the gut–liver axis in BPA toxicity and demonstrate that EOMC exerts a multifactorial protective effect by modulating oxidative stress, microbial metabolism, and xenobiotic elimination. Further mechanistic studies, including transcriptomic and enzymatic analysis, would be useful to better understand the molecular targets of EOMC in detoxification pathways.

## Funding

No funding was received for this manuscript.

## Ethics Statement

All animal care and handling procedures were conducted in accordance with the NIH guidelines and the local Ethics Committee of Tunis University. The protocol was approved by the “Comitéd′Éthique Bio‐médicale (CEBM)” (JORT472001) of the “Institut Pasteur de Tunis.”

## Conflicts of Interest

The authors declare no conflicts of interest.

## Data Availability

The data that support the findings of this study are available from the corresponding author upon reasonable request.
